# Autophagic flux data in differentiated C2C12 myotubes following exposure to acetylcholine and caffeine

**DOI:** 10.1016/j.dib.2016.03.008

**Published:** 2016-03-10

**Authors:** Darin Bloemberg, Joe Quadrilatero

**Affiliations:** Department of Kinesiology, University of Waterloo, Waterloo, Ontario, Canada

**Keywords:** Skeletal muscle, Autophagy, Acetylcholine, Calcium, Caffeine, Myotubes, C2C12

## Abstract

The C2C12 line of mouse myoblasts is a useful cell culture model in which to conduct in vitro analyses related to skeletal muscle. Here we present data regarding the autophagic response induced by two chemicals known to influence calcium release and contraction in skeletal muscles and C2C12 cells: acetylcholine and caffeine. More specifically, by concurrently administering acetylcholine or caffeine along with chloroquine to differentiated myotubes for various amounts of time and assessing the protein expression of LC3 and p62, we report data on the relative level of autophagic flux induced by these two calcium- and contraction-regulating chemicals.

**Specifications Table**

**Table:** 

Subject area	*Biology*
More specific subject area	*Skeletal muscle, autophagy, calcium*
Type of data	*Graphs and Figures*
How data was acquired	*Immunoblotting*
Data format	*Analyzed*
Experimental factors	*Differentiated C2C12 cells were administered acetylcholine or caffeine*
Experimental features	*Fully differentiated C2C12 myotubes were incubated with either acetylcholine or caffeine along with the autophagy inhibitor chloroquine for various times. Cells were then prepared for immunoblot assessment of the autophagy markers LC3 and p62 in order to examine the amount of autophagic flux induced by acetylcholine or caffeine.*
Data source location	*University of Waterloo, Waterloo, Ontario, Canada*
Data accessibility	*All data are provided with this article*

## Value of the data

• Provides data on autophagic flux in differentiated C2C12 myotubes in response to acetylcholine or caffeine.
• Provides data regarding the relationship between skeletal muscle contractile signals and autophagy.
• Help researchers design experiments examining autophagic responses to contraction-related stimuli.

## 1 Data

C2C12 mouse myoblasts are used to perform in vitro analyses related to skeletal muscle as they express proteins involved with membrane depolarization, calcium (Ca^2+^) storage and release, and contraction [1], [2]. Previously, it was shown that Ca^2+^ depletion prevented starvation-induced autophagy in cardiomyocytes [3]. In this report we present data on the degree of autophagic flux in differentiated C2C12 myotubes in response to acetylcholine and caffeine (two stimuli that can influence Ca^2+^ signaling and contraction).

### 1.1 Effect of acetylcholine on autophagic flux in C2C12 myotubes

**Fig. 1:**
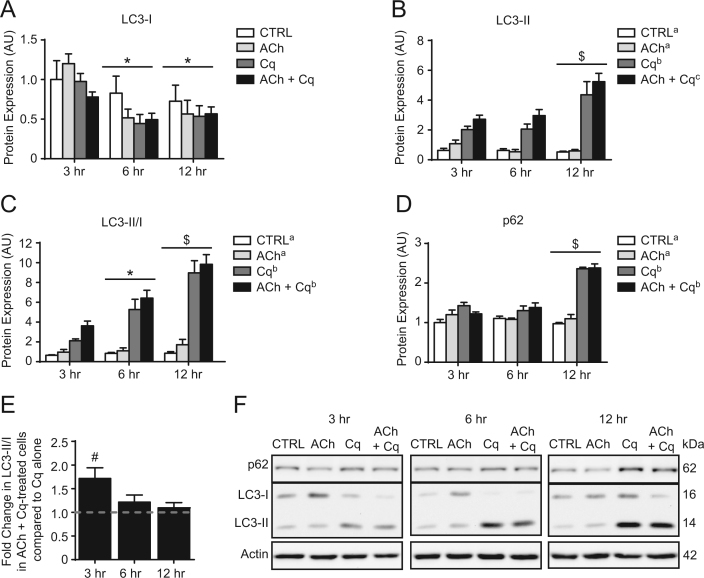
Effect of acetylcholine on autophagic flux in C2C12 myotubes. Protein expression of LC3-I (A), LC3-II (B), and the LC3-II/I ratio (C), where all LC3-I and LC3-II values are expressed relative to CTRL 3 h LC3-I. (D) Relative protein expression of p62. (E) Difference in the LC3-II/I ratio between cells administered ACh+Cq compared to Cq alone. (F) Representative immunoblots of LC3-I, LC3-II, p62, and actin (corresponding to quantitative data presented in panels A, B, C, and D). ^*^p<0.05, significantly different than 3 h (main effect of time); ^$^*p*<0.05 significantly different than 3 h and 6 h (main effect of time). Main effects (*p*<0.05) for treatments are indicated with superscript letters, where groups with different letters are significantly different than each other. ^#^*p*<0.05, significant difference between ACh+Cq and Cq (represented by dashed line) at that time point.

Previous experiments involving acetylcholine (ACh) administration to C2C12 cells have employed a large range of concentrations, from 10 nM to 1 mM [4], [5], [6], [7]. However, we decided to apply ACh at 10 µM, a dose previously shown to cause membrane depolarization, Ca^2+^release, and glucose uptake [4], [5], [7]. Data regarding the induction of autophagy in response to ACh is presented in Fig. 1. As expected, chloroquine (Cq) treatment inhibited autophagic flux as indicated by elevated (*p*<0.05) p62 protein content and the LC3-II/I ratio; however, the addition of ACh did not affect either of these markers (Fig. 1C, D, & F). Although, LC3-II content was increased (*p*<0.05) in ACh+Cq cells compared to Cq alone (Fig. 1B). Most importantly, analyses performed at individual time points showed that at 3 h, the LC3-II/I ratio was 72% higher (*p*<0.05) in ACh+Cq treated cells compared to cells given Cq alone (Fig. 1E). This indicates that 10 µM ACh induced a 72% increase in the amount of autophagy that occurs in cells growing in regular/untreated culture media during this time (Fig. 1E). Although, similar measures at later time points were not significant (*p*>0.05, Fig. 1E), suggesting the effects of ACh are relatively short-lived.

### 1.2 Effect of caffeine on autophagic flux in C2C12 myotubes

**Fig. 2:**
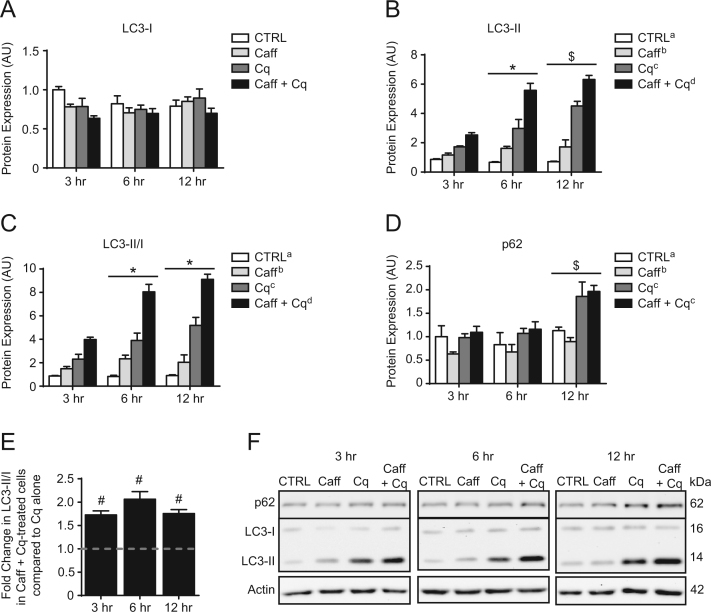
Effect of caffeine on autophagic flux in C2C12 myotubes. Protein expression of LC3-I (A), LC3-II (B), and the LC3-II/I ratio (C), where all LC3-I and LC3-II values are expressed relative to CTRL 3 h LC3-I. (D) Relative protein expression of p62. (E) Difference in the LC3-II/I ratio between cells administered Caff+Cq compared to Cq alone. (F) Representative immunoblots of LC3-I, LC3-II, p62, and actin (corresponding to quantitative data presented in panels A, B, C, and D). ^*^p<0.05, significantly different than 3 h (main effect of time); ^$^*p*<0.05 significantly different than 3 h and 6 h (main effect of time). Main effects (*p*<0.05) for treatments are indicated with superscript letters, where groups with different letters are significantly different than each other. ^#^*p*<0.05, significant difference between Caff+Cq and Cq (represented by dashed line) at that time point.

Caffeine (Caff) was administered to C2C12 myotubes at 2.5 mM, a concentration previously employed by others [8]. Data regarding the induction of autophagy in response to Caff is presented in Fig. 2. Longer treatments were associated with increased (*p*<0.05) accumulation of p62 and LC3-II due to Cq-induced inhibition of autophagy (Fig. 2B, C, D & F). Caff also affected p62 degradation and LC3 lipidation, as Caff independently decreased (*p*<0.05) p62 content (Fig. 2D & F), and additionally increased (*p*<0.05) LC3-II levels as well as the LC3-II/I ratio when applied alone or with Cq (Fig. 2B, C & F). Importantly, the LC3-II/I ratio in Caff+Cq-treated cells was higher (*p*<0.05) at every analyzed time point compared to cells given Cq alone (Fig. 2E). Here, cells treated with Caff for 6 h experienced 2-fold more autophagic flux than cells growing in regular/untreated culture media (Fig. 2E).

## 2 Experimental design, materials and methods

### 2.1 Cell culture and experiment

C2C12 mouse skeletal myoblasts (ATCC) were cultured on polystyrene cell culture plates (BD Biosciences) with growth media (GM) consisting of low-glucose DMEM (ThermoFisher) with 10% fetal bovine serum (FBS; ThermoFisher) and 1% penicillin/streptomycin (ThermoFisher). Upon reaching 80–90% confluence, cells were induced to differentiate by replacing GM with differentiation media (DM) consisting of DMEM with 2% horse serum (ThermoFisher) [9], [10]. Fresh DM was replaced each day. On the 5th day of differentiation, cells were treated with either 10 µM acetylcholine (ACh, Sigma Aldrich), 30 µM chloroquine (Cq, Sigma Aldrich), both ACh and Cq, or DM alone (CTRL) to assess the relative level of autophagic flux [10]. A separate experiment examining the effects of 2.5 mM caffeine (Caff, BioShop) was performed similarly. Acetylcholine, caffeine, and chloroquine were always dissolved on the same day experiments were conducted.

### 2.2 Preparation of cell lysates

Cells were collected via trypsinization at the given time points, centrifuged at 1000g for 5 min, and stored at −80 °C. Cell lysates were prepared for immunoblotting by sonicating cells in lysis buffer containing 20 mM HEPES, 10 mM NaCl, 1.5 mM MgCl, 1 mM DTT, 20% glycerol, 0.1% Triton-X100, and protease inhibitors (Complete Cocktail, Roche) at a pH of 7.4. Protein content of cell lysates was determined using the BCA protein assay method [9].

### 2.3 Immunoblotting

Samples with equal amounts of protein were separated using SDS/PAGE before being transferred onto PVDF membranes [9], [11]. Membranes were incubated with primary antibodies against LC3B (Cell Signaling), p62 (Progen), or actin (Sigma Aldrich) overnight at 4 °C followed by the appropriate horseradish peroxidase (HRP)-conjugated secondary antibody (Santa Cruz) for 1  h at room temperature. Bands were visualized using the Clarity ECL blotting substrate (Bio-Rad) and the ChemiGenius 2 Bio-Imaging System (Syngene). The approximate molecular weight for each band was estimated using Precision Plus Protein WesternC Standards and Precision Protein Strep-Tactin HRP Conjugate (Bio-Rad).

### 2.4 Statistics

Results are presented as means±SEM, where *n*=3 independent experiments performed in duplicate. Comparisons of protein expression across time and between treatment groups were made using 2-way ANOVAs and Tukey post-hoc analyses. Student׳s *t*-tests were used to perform the analyses presented in Figs. 1E and 2E (Ach/Caff+Cq vs Cq). For all analyses, *p*<0.05 was considered statistically significant.
